# Ice Sintering by Sublimation and Condensation

**DOI:** 10.1021/acs.jpclett.5c00050

**Published:** 2025-02-20

**Authors:** Menno Demmenie, Sander Woutersen, Daniel Bonn

**Affiliations:** † Institute of Physics, University of Amsterdam, Science Park 904, 1098 XH Amsterdam, The Netherlands; ‡ Van ’t Hoff Institute for Molecular Sciences, University of Amsterdam, Science Park 904, 1098 XH Amsterdam, The Netherlands

## Abstract

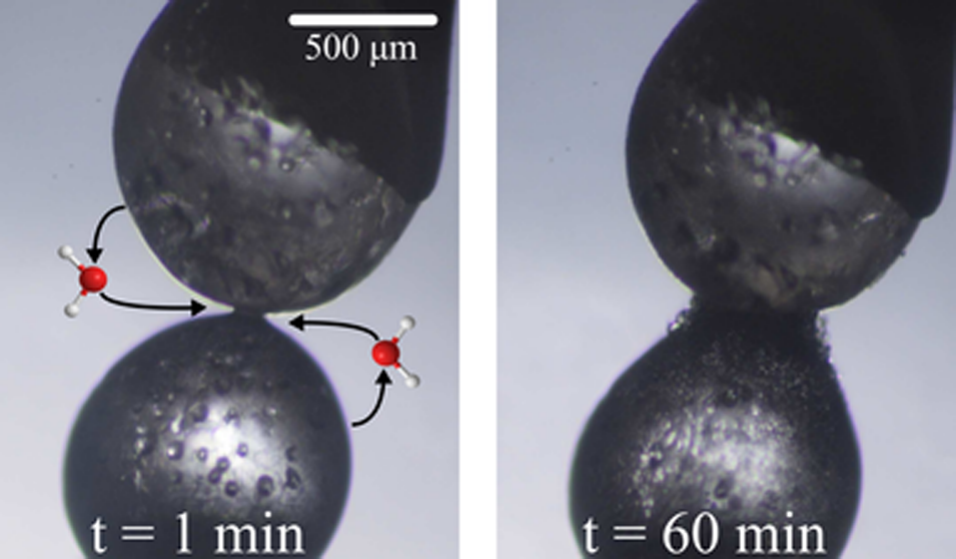

The sintering behavior of ice has been the subject of controversy for more than 160 years. Various factors have led to confusion about the mechanisms behind mass transport during sintering; erroneously derived growth rate exponents, experimental challenges in achieving equilibrium conditions, and incorrect comparisons between ice sintering and snow densification have all played a role. Here we demonstrate that sintering of ice under equilibrium conditions proceeds primarily through sublimation and condensation. Mass transfer occurs through the vapor phase, driven by increased volatility at the formed neck due to its high curvature. Our findings on the sintering of ice spheres are consistent with the healing of micrometer-sized scratches in ice under similar conditions.

More than 160 years ago, Faraday discussed a remarkable feature of ice during one of his famous Christmas lectures: “Two pieces of thawing ice, if put together, adhere and become one; at a place where liquefaction was proceeding, congelation occurs”.^1^ For nearly a century, this hypothesis of Faraday was considered to be valid; it was believed that ice crystals merge due to the presence of a liquid layer that refreezes when enclosed by ice.

In the second half of the 20th century, extensive research on this phenomenon was conducted, known as sintering, which held significant interest in metallurgy.^2−7^ When a heated crystal is placed within a closed system but kept below its melting temperature (*T*_m_), it naturally tends to alter its shape to minimize the surface energy. This evolution in shape can be driven by several mechanisms that facilitate mass transport, including a viscous flow, sublimation and condensation, volume diffusion, and surface diffusion (see Figure 1a). Each of these processes plays a role in reducing the system’s overall energy by reshaping the crystal surface; however, under specific environmental conditions, one mechanism will typically dominate.

**Figure 1:**
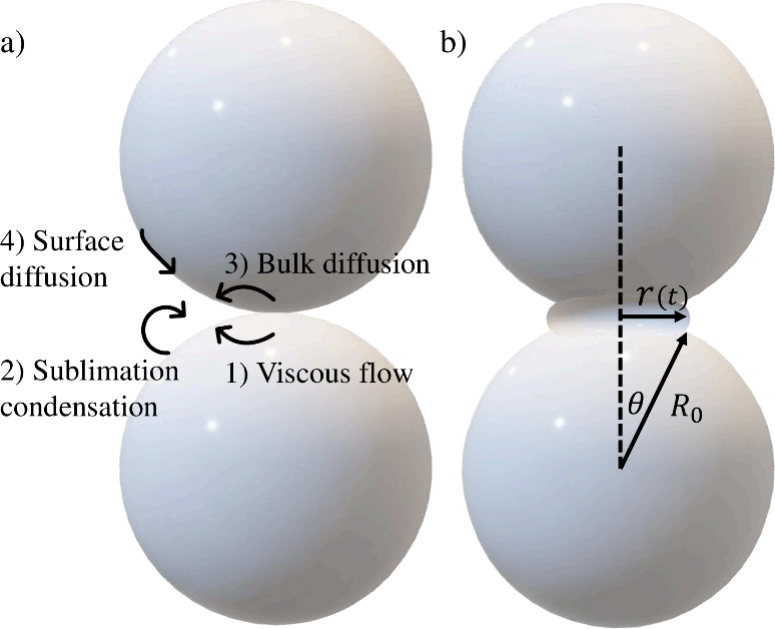
Schematic representation of the four primary sintering mechanisms, labeled by numbers corresponding to *n* in panel a, along with the geometrical parameters used throughout this text, as shown in panel b. *R*_0_ is the initial radius of the sintering sphere, and *r*(*t*) the radius of the neck. Then, θ represents the angle between *R*_0_ projected onto *r*(*t*) and the imaginary (dotted) line between the center points of both spheres.

Experiments on sintering ice spheres, conducted primarily from the 1960s onward, have measured the growth rate of the neck connection of the two spheres as *r* ∼ *t*^α^, and different experiments reported values of α ranging between ^1^/_3_ and ^1^/_7_. While these findings rule out Faraday’s assumption of a liquid layer [for which the growth should be linear (see below)], they raised more questions than they answered about the underlying mechanisms.^4,8−11^ The confusion surrounding the different sintering rates of ice has at least partly resulted from erroneous comparisons with the sintering of snow.^12−14^ Although both ice and snow are composed of crystalline water, their structures differ significantly. Snow densifies on time scales similar to those of ice sintering; the open structure of snowflakes allows gravity to exert sufficient external pressure to cause densification. Thereby, multiple regimes have been revealed from snow to firn to ice, complicating growth rate analyses.^3,15−18^ Furthermore, this violates the fundamental assumption underlying the derivation of growth rates, that the process is exclusively governed by surface minimization and so should not be applied to snow sintering.

Experiments conducted in cold rooms often assume uniform equilibrium conditions but typically lack adequate control over humidity during the experiments. A notable example is the work of Kingery, who reported an exponent α of ∼^1^/_7_,^8^ which was long considered indicative of the correct mechanism. We show in the discussion that this outcome resulted from an underestimation of the relative humidity.

Let us consider the simplest geometrical configuration, two identical crystalline spheres in contact without external pressure. At the point of contact, mass transfers between the spheres, leading to the growth of a so-called neck. The rate of this neck growth over time is dependent on the specific mass transfer mechanism involved. Researchers have attempted to determine particular neck growth rates trough various approaches, including scaling laws^19^ and analytical,^20−24^ numerical,^25−27^ statistical,^28^ and even phenomenological methods.^29,30^ This resulted in a wide range of reported growth rates, some of which are supported by experimental findings. Below, we briefly outline the physics underlying the four primary mechanisms that correspond to distinct growth rates. The integers *n* correspond to the numbering in Figure 1a.

*n* = 1. *Viscous Flow*. For decades, the relation between time *t* and neck radius *r* was incorrectly interpreted as quadratic. This misconception originated from a derivation of Frenkel, which failed to account for mass conservation and incorrectly assumed energy dissipation throughout the entire sintering sphere rather than locally at the neck.^31^ By performing a simple force balance between the capillary force and viscous force (γ ∼ *ηv*, with neck growth velocity ), one would find linear neck growth over time. This is almost correct; although an additional logarithmic time contribution has been theoretically derived,^32,33^ the slight difference in slope between *t* and *r* makes experimental verification challenging. Thus, for the sake of simplicity, we assume linearity *t* ∼ *r*.

*n* = 2. *Sublimation and Condensation*. Sometimes termed mean curvature flow, this mechanism is driven by surface curvature, which increases the vapor pressure of molecules according to the Kelvin equation. In classical gas theory, the rate of molecule emission is directly related to the vapor pressure and, consequently, the surface curvature. At the junction, the rate of condensation must match the rate of the volume change, resulting in a differential equation. Solving this for a spherical geometry with a small neck, so that  with , one would find *t* ∼ *r*^3^. This small neck approximation will also apply to the following two mechanisms.

*n* = 3. *Bulk Diffusion*. At the formation of the neck, where the radius is the smallest, the surface energy is maximized. Reducing the surface area at this point increases the local interfacial volume, achieved through an increase in the number of vacancies within the local lattice structure near the neck. By applying a curvature-dependent vacancy gradient in Fick’s law and integrating, one would obtain *t* ∼ *r*^5^.

*n* = 4. *Surface Diffusion*. This mechanism combines aspects of bulk diffusion and sublimation–condensation, leading to a modified form of Fick’s equation. The curvature at the neck creates a gradient in chemical potential, which in turn drives a net drift in average surface velocity, as described by the Nernst–Einstein equation.^24^ Integrating the resulting differential equation yields *t* ∼ *r*^7^. Simulations by Eggers demonstrated that, during neck growth, a sequence of toroidal voids forms, which must eventually close off, introducing an additional term *t* ∼ r^7^ – r_void_.^34^ Similarly to viscous sintering, we may disregard this additional term for the sake of simplicity due to its minor impact.

Thence, the complete expression for neck growth can be described for all mechanisms by the following equation:
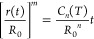
1where *r*(*t*) is the radius of the neck, *R*_0_ is the radius of the initial crystalline sphere, *m* and *n* are mechanism-dependent exponents, and *C*_*n*_(*T*) is a prefactor depending on material properties (for details, see the last column of Table 1).

**Table 1:** Sintering Mechanisms with Corresponding Powers of Neck Growth over Time for a Spherical Geometry, According the Stated Relation in eq 1^a^

mechanism	*n*	*m*	*C*_*n*_(*T*)
viscous flow	1	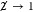	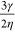
sublimation and condensation	2	3	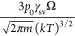
bulk diffusion	3	5	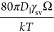
surface diffusion	4	7	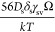

a The most common mistake in the literature is exponent *m* for the viscous flow mechanism; it should be regarded as 1, not 2. The prefactor [*C*_*n*_(*T*)] depends on parameters such as surface tension (γ), viscosity (η), molecular volume (Ω), pressure (*P*_0_), diffusion coefficient (*D*_i_), diffusion surface thickness (δ_s_), Boltzmann constant (*k*), and temperature (*T*).

Leaving prefactors aside, we find the following relation between neck radius *r* and time *t*:
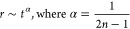
2

To gain new insights into the sintering behavior and the discussion on the anomalies of the outermost layer of ice, we conducted experiments on sintering ice spheres in a humidity-controlled environment.

In these experiments, the spheres were arranged in a horizontal configuration and made contact without external pressure. Establishing an equilibrium vapor pressure in the surrounding box proved to be crucial, as any deviation from the calculated equilibrium value resulted in the observed sublimation or condensation of the entire system. The air temperature in the box, far from the ice, was above *T*_m_. We employed parameters provided by Murphy and Koop to calculate the relative humidity, ensuring that the water vapor pressure within the box corresponds to the saturation vapor pressure of ice.^35^Figure 2 shows a time lapse of an experiment under equilibrium conditions. Two ice spheres, each with an initial diameter of ∼1 mm at −3 °C, undergo relatively fast sintering in the early stages when the radius of the neck is the smallest. Intuitively, neck growth gradually slows over a period of 2.5 h, as the curvature decreases. Throughout the entire experiment, the total volume and mass of the system remain constant, indicating that the vapor pressure equilibrium is maintained. Entrapped air bubbles, formed from dissolved gas that is expelled upon crystallization, appear as darker spots in the ice.^36,37^ We assume that the presence of entrapped air bubbles or even the formation of pits in the contact area has a minimal effect on the sintering rate, as these features inherently exhibit a high curvature and should therefore heal rapidly. Water droplets were formed using two syringes with a needle diameter of 800 μm, which were brought into contact with a horizontal metal hollow torus that was cooled with a constant flow of cooling agent. Once the droplets, which were still attached to the syringes, were fully frozen, they were carefully brought into contact in the center void of the torus. The experiment was vertically monitored using a Nikon D5300 instrument (Navitar 4X HR Plan Apo Objective) illuminated from below by a parallel light-emitting diode light source (Schott KL 2500 LCD). A schematic of the setup is provided in Figure S1.

**Figure 2:**
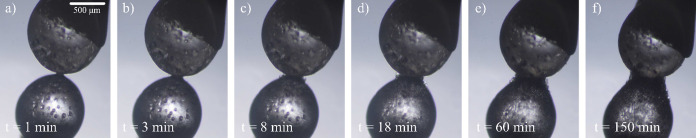
Time lapse of sintering ice spheres at −3 °C with an initial diameter of ∼1 mm. Images were taken from above; i.e., the ice spheres are in contact in a horizontal configuration.

Figure 3 shows the normalized growth of the neck, with respect to the initial sphere diameter (*r*/*R*_0_), over time for four different sintering experiments, conducted under similar conditions. With a simple exponential fit  we obtain values for exponent α of 0.29 ± 0.01, 0.33 ± 0.01, 0.30 ± 0.01, and 0.26 ± 0.01 for the blue, green, orange, and red data, respectively. Here, *t*_0_ represents an experimental delay between the initial contact of the spheres and the first image. Errors were determined by adjusting *t*_0_ by one frame (±15 s). The obtained exponents are in good agreement with α ∼ ^1^/_3_, indicating *n* = 2 and thus representing sublimation and condensation as the mass transport mechanism. When we deliberately set the humidity too low and let the ice sublimate within 2.5 h, the competition between neck growth and sublimation resulted in an exponent of ∼^1^/_7_ (see Figure S2). This highlights the importance of maintaining equilibrium conditions in ice research as the absence of such conditions could account for the variability in reported mass transfer rates over the past several decades.

**Figure 3:**
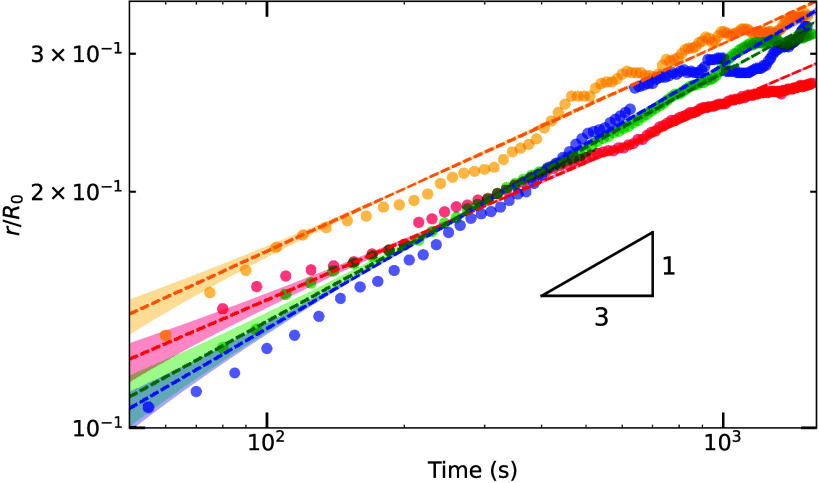
Four different sintering experiments conducted at vapor pressure equilibrium without external pressure at −3 °C. Fits of the normalized neck diameter, with respect to the initial sphere diameter (*r*/*R*_0_) in time, gave values of exponent α of 0.29 ± 0.01, 0.33 ± 0.01, 0.30 ± 0.01, and 0.26 ± 0.01 for the blue, green, orange, and red data, respectively.

Mullins and co-workers showed that the evolution of a profile consisting of a single sine-wave with wavelength λ and amplitude *u* could be described by one differential equation for all previously mentioned mechanisms:^21,38,39^
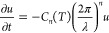
3where the integer *n* again represents the mechanism-dependent exponent, as described in Table 1. They also showed that the equations governing mass diffusion are linear and that the sum of any solution is itself a valid solution. Hence, the evolution of any initial profile can be derived from Fourier analysis. We made a scratch in a pristine ice layer under conditions similar to those to which the the sintering spheres had been exposed and monitored its evolution over time with a confocal profilometer (Keyence VK-X1100).

Figure 4 shows the averaged initial cross section at *t* = 0 in purple dots. This profile was reconstructed with the first 100 Fourier components shown by the purple curve. From there, we let the profile evolve according to eq 3 with the exponent *n* = 2 and with *C*_2_(*T*) as a free fitting parameter. The model, depicted by the colored curves, finds good agreement with the corresponding data points (dots). The inset of Figure 4 shows the initial, non-averaged, profile of the scratch with a length of ∼280 μm and a depth of ∼4 μm that was stretched by 400% for the sake of clarity. Conducting the same procedure for all exponents *n* enabled us to compare the results for sublimation and condensation with those for the other candidate transport mechanisms. Figure 5 shows the evolution of the depth of the scratch in time on a log–log scale along with the four models. Undeniably, the sublimation–condensation model represents the measured data the most accurately. The residual sum of squares yielded values of 0.67, 0.21, 1.93, and 4.55 for *n* values of 1, 2, 3, and 4, respectively. The sublimation–condensation model slightly overestimates the depth on a longer time scale; however, previous research, on the healing of scratches in ice at a wider variety of temperatures, has shown that this is not systematic and that the associated activation energy has excellent agreement with known values for the sublimation of water molecules.^40^ A comparison of the four candidate healing mechanisms, considering the complete profile and not only the depth evolution (Figure S3), highlights the strong agreement with the sublimation–condensation model.

**Figure 4:**
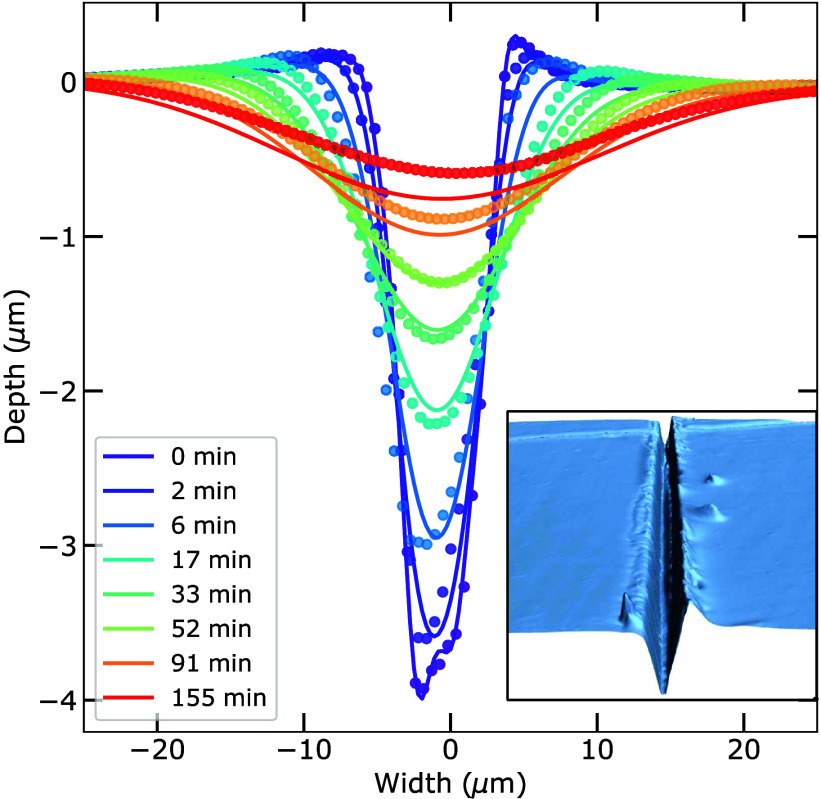
Evolution of a scratch of ∼4 μm deep made in a pristine layer of ice. Dots and curves represent the measured data and model, respectively. The data at *t* = 0 are reconstructed with a Fourier series and evolved over time using eq 3, with *n* = 2 and *C*_2_(*T*) being a free fitting parameter. The inset depicts the initial scratch in three dimensions with a length of ∼280 μm.

**Figure 5:**
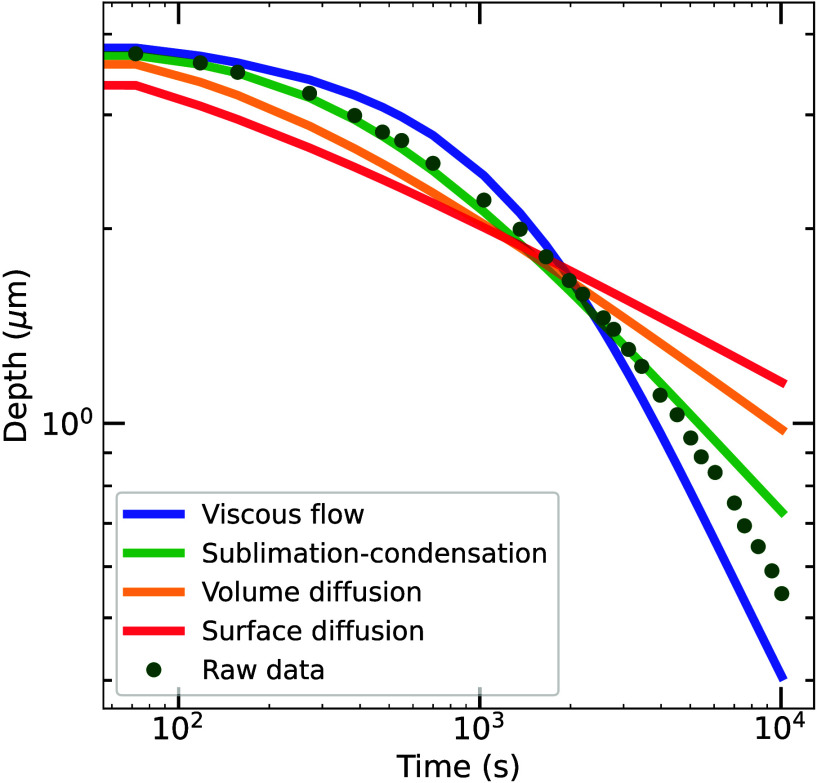
Maximum depth of a scratch healing in ice on a log–log scale. Green data points correspond to the experimental data; the best fits of the four candidate models are shown by the solid lines.

The data, both for sintering spheres and the healing of scratches, support the second mechanism, sublimation and condensation, in which mass transfer occurs through the vapor phase. Previously, Maeno and Ebinuma predicted that vapor phase mass transport would dominate ice sintering, based on calculations of the exact growth rate from parameters in the prefactor.^41^ An exception was identified for neck sizes that were <8% of the initial sphere diameter (*r*/*R*_0_ < 0.08), where surface diffusion becomes predominant within certain temperature ranges. More recently, Chen et al. used SEM imaging to show that sintering of a quasi-one-dimensional stack of ice spheres occurs through ∼10^–4^ m-sized protrusions that bridge the spheres in the downward direction. They concluded that upward vapor transfer caused the downward growth of ice and attributed the protrusion sizes to instable Mullins–Sekerka-type growth.^42^ Their analogy to instable growth from an undercooled melt may be valid but could also be driven by a downward temperature gradient. The pile of spheres was cooled from the bottom, generating a temperature gradient that is difficult to maintain under equilibrium conditions. In contrast, our experiments took place in a humidity-controlled environment with spheres maintained at equal temperatures, which is presumably the reason we do not observe such protrusions.

The surface of ice remains a topic of intense scientific debate with conflicting evidence and interpretations leading to a complex picture. As reviewed by Nagata et al., there are several perspectives on the nature of the ice surface, particularly with regard to the existence and properties of a quasi-liquid layer (QLL).^43^ On one hand, sum-frequency generation (SFG) spectroscopy, combined with molecular dynamics (MD) simulations, suggests that the outermost layer of ice begins to exhibit disorder at temperatures as low as −90 °C, with further disorder occurring in a bilayer-by-bilayer manner as the temperature increases.^43^ Recent advancements in SFG spectroscopy, particularly by Yamaguchi et al., have further probed the structure and dynamics of the air–ice interface, offering deeper insights in the properties of this disordered layer.^44^ Complementary MD simulations provide atomic-scale details of these structural and dynamical changes, supporting the notion of a temperature-dependent transition at the surface.^45^

On the other hand, large-scale MD simulations challenge the idea of a distinct premelting onset temperature. Instead, these simulations indicate a continuous increase in liquid-like water at the surface as the temperature increases, without a discrete temperature threshold for premelting.^46^ This discrepancy highlights the sensitivity of simulation results to factors such as the length and time scales employed. Bartels-Rausch et al. have provided a comprehensive review of air–ice chemical and physical interactions, highlighting the complexity of the QLL and its role in various atmospheric processes.^47^ Furthermore, Pickering et al. have questioned whether the QLL can truly be considered a liquid, using grand canonical investigations to probe its properties.^48^ These diverse perspectives underscore the complexity of ice surface behavior and the need for further research to reconcile different observations and develop a more comprehensive understanding of the ice surface.

However, recent quantitative experiments have shed new light on the nature of the ice surface. Canale et al. obtained complex rheological properties using tuning fork measurements, observing a high viscosity combined with elastic responses.^49^ Weber et al. demonstrated that the mobility of water molecules at the ice surface is remarkably high.^50^ This high mobility could provide an explanation for phenomena such as the slipperiness of ice and its self-healing properties. Building on this work, we previously showed that the apparent fluidity of the ice surface may be attributed to a sublimation–condensation mechanism rather than the presence of a distinct liquid-like layer.^40^ These findings suggest that while the ice surface exhibits high molecular mobility, it may not possess all of the properties typically associated with liquids. The sublimation–condensation mechanism could explain various phenomena associated with ice surfaces without invoking the presence of a liquid layer. This perspective challenges the traditional view of a distinct QLL and highlights the complexity of the ice surface behavior, which cannot be simply categorized as either solid or liquid.

In conclusion, we present strong evidence that sintering of ice spheres, while placed in vapor pressure equilibrium, is driven by local sublimation and condensation. These findings align with experiments on the self-healing behavior of ice. The high vapor pressure, distinctive to ice, arises from the limited cooperativity of hydrogen bonding at the surface and facilitates sublimation from and condensation onto the surface. Thus, we propose that the surface of an ice crystal might be better understood as a rapidly diffusing two-dimensional gas, potentially explaining its anomalous properties.

## Supporting Information Available

Schematic representation of the experimental setup, sintering measurement out of vapor pressure equilibrium, and comparison of the full profile of the four candidate self-healing models (PDF)
