# Supplementary material for: Ice Sintering by Sublimation and Condensation
Source: J Phys Chem Lett. 2025 Feb 20;16(8):2104–9. doi: 10.1021/acs.jpclett.5c00050 (PMC11874037; doi:10.1021/acs.jpclett.5c00050)
Supplement: Supplementary file 1 — jz5c00050_si_001.pdf [file jz5c00050_si_001.pdf]

Ice Sintering by Sublimation and Condensation

Menno Demmenie,[*,†,‡] Sander Woutersen,[‡] and Daniel Bonn[†]

†*Institute of Physics, University of Amsterdam, Science Park 904, 1098 XH Amsterdam, the Netherlands*

‡*Van 't Hoff Institute for Molecular Sciences, University of Amsterdam, Science Park 904, 1098XH Amsterdam, The Netherlands*

E-mail: m.demmenie@uva.nl

Supplemental Material

Fig. S1 provides a schematic representation of the experimental setup used for the sintering experiments. Fig. S2 presents data illustrating the growth of a neck in a system where the humidity was deliberately set too low to observe sublimation. Fig. S3 compares four models for the scratch-healing of a micron-sized scratch in ice ($T_{\mathrm{ice}} \sim 270$ K).

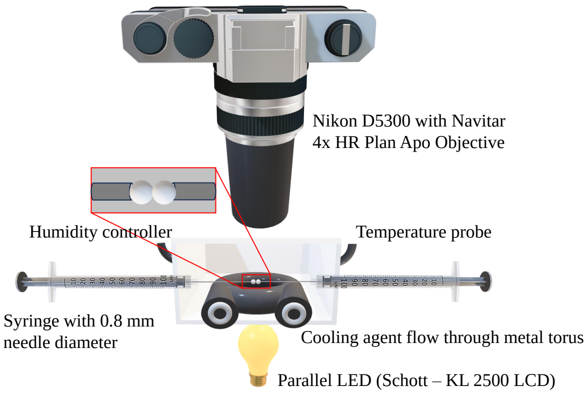

Figure S1: Schematic representation of the used setup.

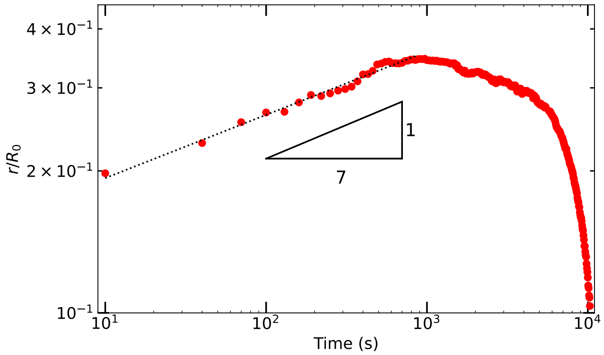

Figure S2: Normalized neck size in time (r/R_0), measured in non-equilibrium conditions. Humidity was deliberately put too low in order to let the entire system sublimate. As a result, the neck grows with a fitted power α of $0.138 \pm 0.01 \sim 1/7$, similar to what Kingery reported.[1]

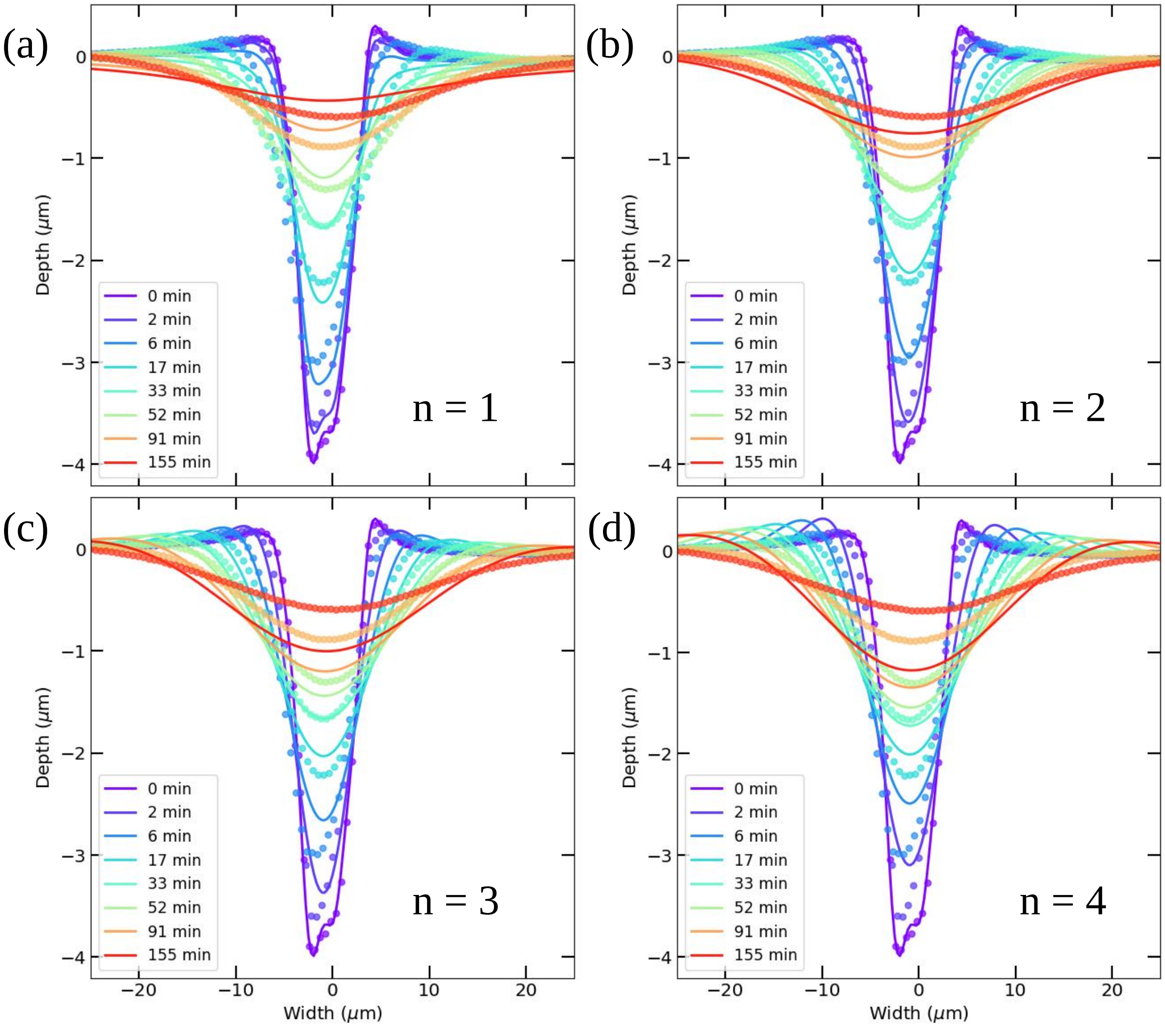

Figure S3: Comparison between the four candidate self-healing models of a micron-sized scratch in ice ($T_{\text{ice}} \sim 270$ K). For each time step, dots depict experimental data, whereas solid lines represent the model of a viscous flow (a), sublimation and condensation (b), volumetric bulk diffusion (c) and surface diffusion (d), respectively.

References

(1) Kingery, W. Regelation, surface diffusion, and ice sintering. *Journal of Applied Physics* **1960**, *31*, 833–838.
